# Draft Genome Sequences of Citrobacter freundii and Citrobacter murliniae Strains Isolated from the Feces of Preterm Infants

**DOI:** 10.1128/MRA.00494-19

**Published:** 2019-08-15

**Authors:** Yuhao Chen, Thomas C. Brook, Cristina Alcon-Giner, Paul Clarke, Lindsay J. Hall, Lesley Hoyles

**Affiliations:** a Department of Surgery and Cancer, Imperial College London, London, United Kingdom; b Department of Biomedical Sciences, University of Westminster, London, United Kingdom; c Gut Microbes and Health Programme, Quadram Institute Bioscience, Norwich, United Kingdom; d Neonatal Intensive Care Unit, Norfolk and Norwich University Hospital, Norwich, United Kingdom; e Department of Biosciences, Nottingham Trent University, Nottingham, United Kingdom; University of Maryland School of Medicine

## Abstract

Here, we describe the draft genome sequences of three strains of Citrobacter isolated from feces of preterm neonates with suspected sepsis. Strains P106E PI and P079F I were Citrobacter freundii. Strain P080C CL represents the first draft genome sequence of Citrobacter murliniae.

## ANNOUNCEMENT

Species of the genus *Citrobacter* are considered members of the human gut microbiota and are opportunistic pathogens in a range of nosocomial infections (1). Worldwide, they are associated with neonatal sepsis in a subset of infants, and multidrug-resistant strains are being detected with increasing frequency (2–6).

**FIG 1:**
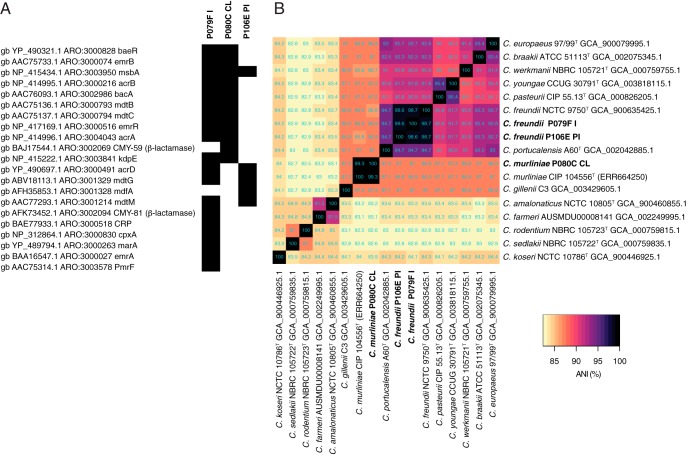
(A) Antibiotic resistance determinant homologs found in the genomes of the three *Citrobacter* strains recovered from the feces of preterm neonates. Antibiotic Resistance Ontology (ARO) annotations were retrieved from Comprehensive Antibiotic Resistance Database (CARD) matches, with only those homologs that gave a strict match with CARD reference sequences based on CARD-recommended bit score cutoffs (gene dependent) for BLASTP analyses included in the figure (black). White, no homologous match. (B) Heatmap showing ANI values obtained with FastANI (15) for representatives of the genus *Citrobacter* and the three neonate strains.

Fecal samples were collected from three preterm neonates with suspected sepsis. Briefly, after storage at −80°C, fecal samples were diluted 1:10 in TBT buffer (100 mM Tris/HCl [pH 8.0], 100 mM NaCl, and 10 mM MgCl_2_ · 6H_2_O) and plated onto MacConkey agar no. 3 and incubated overnight at 37°C to isolate lactose-positive (pink) colonies (7). Details for the sources of the strains described here can be found in Table 1. Phenotypic testing (API 20E) identified the strains as *Citrobacter* sp. DNA was extracted using a phenol-chloroform method described fully by Kiu et al. (8) from overnight cultures of strains and sequenced using the 96-plex Illumina HiSeq 2500 platform to generate 125-bp paired-end reads (9). Raw data provided by the sequencing center were checked using FastQC v0.11.4 (https://www.bioinformatics.babraham.ac.uk/projects/fastqc/); no adapter trimming was required, and reads had an average Phred score of >25. MetaPhlAn2.6 (10) was used to identify the closest relatives of strains, leading to a reference-based (Citrobacter freundii complex strain MGH104; Assembly accession no. GCA_001034485) assembly being produced by BugBuilder v1.0.3b1 (default settings for Illumina assembly) (11). Summary statistics for the genome sequences are given in Table 1, with completeness (99.9, 99.9, and 100%, respectively) determined using CheckM v1.0.13 (12). Genomes were annotated using the NCBI Prokaryotic Genome Annotation Pipeline (13). BLASTP analysis of the proteomes of the three strains against Comprehensive Antibiotic Resistance Database (CARD) data v3.0.1 (https://card.mcmaster.ca/latest/data) (14) using the recommended bit score cutoffs for strict matches (gene dependent) showed the strains to encode a range of antibiotic resistance determinant homologs, with two strains encoding β-lactamases and one encoding PmrF, which is linked to colistin resistance (Fig. 1A).

**TABLE 1:** Clinical information and genome sequence statistics for the three *Citrobacter* strains

Strain	Source of feces	Genome information							
		No. of reads	Size (bp)	No. of contigs	Coverage (×)	*N*_50_ (bp)	No. of CDS^*a*^	No. of tRNAs	G+C content (%)
P079F I	12-day-old male; Caesarean section (gestational age, 30 wks); wt, 1,544 g	989,778	5,273,335	64	47	261,533	5,056	71	51.8
P080C CL	12-day-old male; vaginal delivery (gestational age, 25 wks, 5 days); wt, 831 g	1,132,580	5,024,923	59	56	260,081	4,647	69	50.6
P106E PI	10-day-old female; vaginal delivery (gestational age, 30 wks, 4 days); wt, 1,402 g	1,149,416	5,139,193	106	56	178,284	4,840	72	51.3

a CDS, coding sequences.

FastANI (15) was used to determine the average nucleotide identity (ANI) of the genomes against that of the type strain, NCTC 9750^T^, of C. freundii (Assembly accession no. GCA_900635425). P106E PI and P079F I were confirmed to be Citrobacter freundii (98.6% and 98.7% ANI, respectively) (16–18). Multilocus sequence typing showed P079F I to be sequence type 311 (ST311) and P106E PI to be ST95. Strain P080C CL was assigned as a *Citrobacter* sp. by MetaPhlAn2.6, so its 16S rRNA gene sequence was identified within the whole-genome sequence using RNAmmer v1.2 (19) and compared against 16S rRNA gene sequences available at EzBioCloud (https://www.ezbiocloud.net/) (20). It shared 100% similarity with Citrobacter murliniae CDC2970-59^T^. To determine whether P080C CL represented a strain of C. murliniae, sequence reads deposited for the type strain, CIP 104556 (1), were downloaded from the Sequence Read Archive (accession no. ERR664250) and assembled using SPAdes v3.11.1 (default settings) (21) for inclusion in ANI analyses (Fig. 1B). Strain P080C CL shared 99.3% ANI with *C. murliniae* CIP 104556^T^ and is therefore a representative and first available draft genome sequence of this species (16–18).

### Data availability.

These whole-genome shotgun projects have been deposited in DDBJ/ENA/GenBank under the accession no. QFTZ00000000 (P079F I), QFVP00000000 (P080C CL), and QFTQ00000000 (P106E PI). Raw data have been deposited under accession no. SRR9048465, SRR9048466, and SRR9048464, respectively. The versions described in this paper are the first versions, QFTZ01000000, QFVP01000000, and QFTQ01000000, respectively.
